# Open Agile text mining for bioinformatics: the PubAnnotation ecosystem

**DOI:** 10.1093/bioinformatics/btz227

**Published:** 2019-04-01

**Authors:** Jin-Dong Kim, Yue Wang, Toyofumi Fujiwara, Shujiro Okuda, Tiffany J Callahan, K Bretonnel Cohen

**Affiliations:** 1 Database Center for Life Science, Research Organization of Information and Systems, Kashiwa, Chiba, Japan; 2 Graduate School of Medical and Dental Sciences, Niigata University, Niigata, Japan; 3 Computational Bioscience Program, University of Colorado Denver, Anschutz Medical Campus, Aurora, CO, USA; 4 Université Paris-Saclay, LIMSI-ILES, France

## Abstract

**Motivation:**

Most currently available text mining tools share two characteristics that make them less than optimal for use by biomedical researchers: they require extensive specialist skills in natural language processing and they were built on the assumption that they should optimize global performance metrics on representative datasets. This is a problem because most end-users are not natural language processing specialists and because biomedical researchers often care less about global metrics like F-measure or representative datasets than they do about more granular metrics such as precision and recall on their own *specialized* datasets. Thus, there are fundamental mismatches between the assumptions of much text mining work and the preferences of potential end-users.

**Results:**

This article introduces the concept of *Agile text mining*, and presents the *PubAnnotation ecosystem* as an example implementation. The system approaches the problems from two perspectives: it allows the reformulation of text mining by biomedical researchers from the task of assembling a complete system to the task of retrieving warehoused annotations, and it makes it possible to do very targeted customization of the pre-existing system to address specific end-user requirements. Two use cases are presented: assisted curation of the *GlycoEpitope* database, and assessing coverage in the literature of pre-eclampsia-associated genes.

**Availability and implementation:**

The three tools that make up the ecosystem, *PubAnnotation*, *PubDictionaries* and *TextAE* are publicly available as web services, and also as open source projects. The dictionaries and the annotation datasets associated with the use cases are all publicly available through PubDictionaries and PubAnnotation, respectively.

## 1 Introduction

These are exciting times for biomedical research, and that excitement is reflected in the volume of publications available in PubMed/MEDLINE (http://www.pubmed.gov/), the US National Library of Medicine’s database of biomedical publications—at the time of writing, contained 28 million articles. But, that very volume creates a problem for researchers: staying on top of relevant literature is important, as is targeted reading in order to interpret results and evaluate potential follow-ups to experimental findings. Unfortunately, the enormous volume of publications can make digesting even the most relevant literature in a typical scientist’s field essentially impossible. Consider e.g. (Alper *et al.*, 2004) calculated that physicians trained in epidemiology would need an estimated 627.5 h per month to keep up with the journals required for primary care work in their field.

Text mining has been suggested as a solution for managing the overabundance of published research, and judging from the amount of work in the area, there seems to be a consensus that it has significant potential (Altman *et al.*, 2008; Jovanovi and Bagheri, 2017). However, it remains unclear as to whether existing text mining tools are usable by biomedical scientists: they typically require very specific technical skills that are outside of the normal training of researchers in other fields (Wang *et al.*, 2016). A number of approaches have been taken to improve the accessibility of text mining tools to that target audience. One family of approaches has focused on building wrappers around text mining functionalities in order to make it possible for non-specialists to become acquainted with an integrated text mining framework, rather than having to use many disparate tools at different stages of the analysis (Batista-Navarro *et al.*, 2016; Rak *et al.*, 2012, 2014; Roeder *et al.*, 2010; Yoshinobu *et al.*, 2009, 2011). Other families of approaches have focused on usability, building interfaces that are meant to provide graphical user interface-like tools for constructing a conceptually clear-cut text mining pipeline (Condie and Urbanski, 2014; Garten and Altman, 2009; Müller *et al.*, 2004). Although the uptake of tools like Textpresso within the model organism database community (see the previous citations) has been impressive, adoption of these tools and the associated text mining technology by bench scientists has been negligible—it is notable that usability issues for text mining tools were pointed out as early as ten years ago (Altman *et al.*, 2008). All of these approaches have something in common: they focus on addressing the problem of end-user access to text mining by making it easier for users to interact with the tools. In contrast, the approach that is taken here is to give the user more control of the entire process: defining what to annotate, generating annotations, examining outputs and responding to necessary changes. From the perspective of software engineering theory, this suggests modeling the work as an Agile methodology, and as we show in the Discussion section, there is good evidence that this can be a fruitful approach in bioinformatics (Mishima *et al.*, 2011; Silva *et al.*, 2016; Su *et al.*, 2010), but that it has been under-explored in text mining. 

For context: a researcher’s first strategy for solving a text mining problem might be to identify readily available and/or easy-to-implement text mining resources [e.g. *MetaMap* (Aronson *et al.*, 2010), *PubTator* (Wei *et al.*, 2013) or *Tagger* (Jensen, 2017)]. What about situations where a text mining problem that cannot readily be solved with existing resources? Specific text mining needs are likely diverse, potentially corresponding to a wide variety of biological or bioinformatic interests, an observation partly reflected in the variety and size of available biomedical ontologies or terminologies (e.g. MeSH, Open Biomedical Ontologies).

Based on these observations, we draw the conclusion that *customization* is an important but neglected feature of text mining systems; biologists or bioinformaticians, regardless of their technical expertise, should easily be able to personalize the annotation process to fulfill their specific needs. To account for this idea, we propose adopting the concept of *Agile text mining* (The term is motivated by the term *Agile software development*, which advocates the ability to respond to the change of needs; Beck *et al.*, 2001), for which (i) there should be explicit ways to specify the system parameters in order to generate the desired output, and (ii) once the specification is ready, retrieval of results should be trivial. As a proof of concept, we present PubAnnotation, an ecosystem which enables Agile text mining, and focus on the case of dictionary-based text annotation, which is fundamental to the universal task of text mining.

## 2 Materials and methods

Agile methods, in general, focus heavily on adaptive response to user needs. In biomedical text mining, *named entity recognition and normalization* or the identification of biologically and clinically relevant concepts in text, such as genes and gene products, diseases and treatments, is—and has long been, continuing to the time of writing—one of the most common applications (Cohen and Demner-Fushman, 2014; Hirschman *et al.*, 2012; Nguyen *et al.*, 2019; Tanabe and Wilbur, 2002). Because of that prevalence of named entity recognition as a user-defined need, we describe in depth its exploration as a challenge for Agile text mining. Dictionary-based text annotation is one of the primary methods for making the PubAnnotation ecosystem adaptable by end-users. Thus, before providing an overview of the ecosystem design principles and components (i.e. *PubAnnotation*, *PubDictionaries* and *TextAE*), a brief introduction to dictionary-based text annotation is provided.

### 2.1 Dictionary-based annotation of text

A common approach to both named entity recognition and normalization involves using a ‘dictionary’, or a set of identifiers associated to strings with which they can be mentioned in text. For example, the Pre-eclampsia ontology (Mizuno *et al.*, 2016) contains the SNOMEDCT_15938005 (merged with SNOMED CT), which has six different strings associated with it, including *Eclampsia, toxemia with convulsions, Eclamptic toxemia, Toxemia with convulsions, Eclamptic toxemia, Toxemia with convulsions and Eclampsia (disorder)*.

Although there is considerable variability in today’s approaches to normalization in biomedical text, dictionary-based approaches remain popular, with publications based entirely or in a large part on dictionaries just in the past year (Chen *et al.*, 2017; Duz *et al.*, 2017; Dziadek *et al.*, 2017; Garten *et al.*, 2018; Gipson *et al.*, 2017; Kasthurirathne *et al.*, 2016; Patterson *et al.*, 2017; Pierce *et al.*, 2017). This is somewhat surprising, since there is a long history of critiques of dictionary-based named entity recognition and normalization, dating back at least to the first shared task on gene name recognition (Yeh *et al.*, 2005). However, their ability to scale across many domains without requiring training data has kept them popular, and although they may require considerable tuning, they are capable of high performance (see, e.g. the evaluation of multiple systems in Funk *et al.* 2014).

Specification of a dictionary-based text annotation task may consist of: (i) a dictionary, specifying a set of entities, and (ii) a set of target texts. A desired result of the task would be the index of the dictionary entities corresponding to referenced target texts.

As a simple example, a dictionary for Pre-eclampsia, a hypertensive disorder of pregnant women that affects from 2 to 8% of all pregnancies world-wide (Sibai *et al.*, 2005), might have two entries:

- (*Pre-eclampsia*, ORPHA: 275555)
- (*pre-eclamptic toxemia*, ORPHA: 275555)

For the input *Individual blood differences in relation to pregnancy, with special reference to the pathogenesis of pre-eclamptic toxemia.* (PMID: 13184842), the desired output would then be:

- (PubMed: 13184842, 35-56, ORPHA: 275555)

where the number range of 35–56, represents the index of the location in the text (i.e. PubMed entry 13184842), in character offsets, where *PE* is referred to: a *text-anchored annotation*.

Useful implementations of dictionary-based text annotation often expand exact string matching to include features like stemming and disambiguation (Jensen, 2017). To account for these scenarios, the specification may include parameters to specify a *matching criterion*. Assuming that the quality of the result of a dictionary-based text annotation is related to the dictionary and the matching criterion, and that the performance can be empirically evaluated by manually checking the annotation results, a dictionary-based text annotation workflow would include:

1. Initial preparation of a dictionary and a matching criterion.
2. An iterative process for improving the dictionary and the matching criterion while testing performance.
3. Production of results using the final dictionary and matching criterion.

Note that if there is a benchmark dataset (i.e. *gold standard* or *gold annotation*), Step 2 of the workflow could be automated. In many cases, a gold-standard dataset is not available; furthermore, end-users may have very particular performance requirements, as discussed in Alex *et al.* (2008). Step 2 also can be used to produce a benchmark dataset.

### 2.2 The PubAnnotation ecosystem: design principles

PubAnnotation supports an Agile approach to text mining by instantiating software components that allow for decomposed parallel development, while also facilitating continuous integration. Three components in particular are used to implement the iterations of Agile development:

1. A manual annotation tool allows for the specification of the language processing version of a user story: an annotation model.
2. A dictionary-based annotator allows push-button evaluation.
3. A storage component facilitates regression testing.

Thus, the iterative development/testing cycle of an Agile text mining sprint can be carried out as follows: (i) modify a dictionary, (ii) manually reannotate, (iii) automatically reannotate and benchmark. Additionally, for cases where no gold standard dataset is available for benchmarking, PubAnnotation allows random sampling of the automatically annotated text for manual evaluation.

### 2.3 Implementation details

The PubAnnotation ecosystem is designed to be an open, API-driven system. By leveraging a RESTful API, the ecosystem becomes open for any implementation that conforms to REST standards (i.e. every component of the ecosystem needs to be implemented as a Web service or a Web client in the HTTP network). By this principle, potential users should be able to use the system immediately without having to go through the difficulty of specifying system settings themselves.

**Fig. 1.**
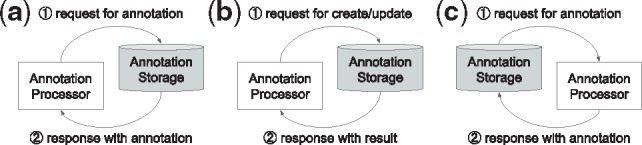
Communication models of PubAnnotation: passive communication model, (**a**) and **(b)**, and active communication model, (**c**)

The PubAnnotation ecosystem is comprised of three of types of annotation components: (i) *Storages* (i.e. static components used to store and maintain annotations); (ii) *Accessors* (i.e. access unaltered annotations from storage); and (iii) *Processors* (i.e. dynamic components used to create or revise annotations). Two communication models have been designed to facilitate communication among the components:

- In the *Passive communication model*, an annotation accessor or a processor, initiates communication to access annotations stored on an annotation server (Fig. 1a), or to push new or revised annotations to a server (Fig. 1b). In this model, an annotation storage either ‘passively’ allows access to annotations or receives annotations, highlighting the differences in nomenclature. We suppose this model is suitable for an accessor (e.g. annotation viewers) or a processor, which involves human intervention (e.g. annotation editors).
- In the *Active communication model*, an annotation storage initiates communication to ‘actively’ obtain new or updated annotations (Fig. 1c). We suppose this model is suitable for automatic annotators, which can immediately respond for the request from the storage.

Based on these two communication models, several components have been developed within the ecosystem, this article presents three: *PubDictionaries*, *PubAnnotation* and *TextAE*. As PubDictionaries and PubAnnotation act as a processor and a storage, respectively, PubAnnotation can obtain annotations from PubDictionaries, through the active communication model. TextAE acts as either an accessor, when it is used as a viewer, or a processor, when it is used as an editor. In either case, the communication is initiated by TextAE, to which PubAnnotation responds, through the passive communication model. We will use these components to demonstrate an ‘Agile’ dictionary-based text annotation and mining workflow, the focus of which is on enabling users to quickly customize and develop a dictionary-based text annotation.

### 2.4 PubDictionaries: a dictionary-based annotator

Although the ecosystem includes various annotation processors (http://pubannotation.org/annotators), e.g. syntactic parsers, and named entity recognizers, PubDictionaries (http://pubdictionaries.org) is a specially-designed one to enable highly customizable dictionary-based annotation, and is of primary focus in this work. PubDictionaries is implemented to provide following functions:

- A repository of dictionaries,
- An interface to test dictionary-based annotation,
- A set of programable RESTful APIs for scalable annotation,
- An interface to enable edition of dictionaries.

As the name implies, PubDictionaries is implemented as a public repository of dictionaries, which means users can register their dictionaries to PubDictionaries. Once a dictionary is registered, it can be used for text annotation, through either a user interface or a set of programable RESTful APIs. Although the user interface enables a quick test of the dictionary for annotation, the programable RESTful APIs enable fast annotation at scale. As the dictionaries for annotation can be chosen among those registered in PubDictionaries, it effectively works as a plug-in system of dictionaries for dictionary-based annotation. PubDictionaries also provides a user interface for dictionary editing: a user can easily add a new entry to or delete an entry from a dictionary. Together with the user interface for testing dictionary-based annotation, it effectively facilitates developing a dictionary in a try-and-revise manner.

Here, a dictionary means a collection of pairs of labels and identifiers:

(1) $$\Delta =\{(l_{1},i_{1}),\ldots ,(l_{k},i_{k})\}$$

where labels mean term expressions, expected to appear in natural language text in order to refer to specific entities, and identifiers are structured information sources (e.g. databases or ontologies).

### 2.5 PubAnnotation: an annotation storage depot

PubAnnotation (http://pubannotation.org) is originally implemented as a persistent and shareable storage of corpus annotation (Kim and Wang, 2012). Since then, its functionality is substantially extended to facilitate production of annotations, and now it supports following tasks which are required to produce high-quality annotations: Preparation of target texts, Automatic annotation, Manual correction and Searching annotation.

To simplify the task of preparing documents for annotation, PubAnnotation implements *text sequencers*, which process documents to make them ready for annotation. Concretely, a text sequencer turns a document into a sequence of characters, so that positions in the document can be specified unambiguously by character offsets. Currently, PubAnnotation has two sequencers for PubMed and PMC.

Once texts for annotation are prepared, annotation may be produced in many ways. Here, we focus on dictionary-based annotation, of which the utility is discussed in Section 2.1, particularly with PubDictionaries. To annotate the texts within a project on PubAnnotation using an annotation processor like PubDictionaries, the user first needs to know the URL of the annotation processor. For example, the URL of the PubDictionaries annotation service that uses the dictionary *Pre-eclampsia* is as follows: http://pubdictionaries.org/text_annotation.json?dictionary=Preeclampsia, to which a block of text can be sent to get annotation to the text. PubAnnotation offers a user interface through which the URL of the annotation service may be specified (PubAnnotation also features an easier point-and-click interface, through which users can choose one of the pre-registered annotation services.). PubAnnotation will then send all the texts within the project to PubDictionaries, either sequentially or in a batch mode, and store the results within the project. The back-end database of PubAnnotation, implemented on PostgreSQL, facilitates a stable and scalable storage for annotations. (The URL can be easily obtained from the web site of PubDictionaries.)

After a set of annotations are produced through an automatic annotation process, the next step would be to evaluate the quality to determine if it is good enough or if it needs improvement. In an ideal situation, there may be a benchmark dataset (a.k.a. *gold standard* or *gold annotation*) prepared for evaluation of the annotation. If this is the case, the benchmark dataset can be registered to PubAnnotation as a project and compared with any other project. PubAnnotation offers an easy interface to do so, and the result is reported in recall, precision and F1-score. More often, however, there may be no benchmark dataset. In the case, a set of annotations may be evaluated through manual inspection. PubAnnotation provides several tools to ease this task, most notably the SPARQL-based search interface, as well as standard sorting and a simple keyword-based search interface powered by Lucene indexing (http://lucene.apache.org/).

To enable the SPARQL-based search interface over a project, the annotations in the project need to be converted to resource description framework (RDF) statements (https://www.w3.org/RDF/), which is a one-click process on PubAnnotation. Once the RDFization is done, SPARQL (https://www.w3.org/TR/sparql11-overview/) queries may be used for searching specific patterns over the annotations, even across multiple projects (e.g. ‘*To find sentences which have a disease annotation from a project and also a gene annotation from another project’*). The search interface plays a similar role to *concordancers* (e.g. software which assists in searching and analyzing text from a corpus), which are proven to be useful for manual inspection of text annotation (Silberztein, 2005; Thomas, 2007).

After evaluation is done either in an automatic or a manual way, if the quality is not satisfactory, users may want to improve the performance. An improvement may be implemented either by revising the dictionary using PubDictionaries, or by revising the annotation using PubAnnotation. In the case of dictionary revision, missing entries and noisy entries, which have been identified during the evaluation, will be added to or deleted from the dictionary, respectively. In this case, the improvement may be replicated to future (dictionary-based) annotations. In the case of annotation revision, the improvement will not be replicated, but the revised annotation may be regarded as the correct one, and this option may be chosen to develop a benchmark dataset of the annotation for future evaluation.

As discussed in the beginning of this section, PubAnnotation works as a public repository of annotations, as well as a platform for annotation development. So, annotations developed on PubAnnotation can be shared. To date, PubAnnotation contains almost 200 projects, making it one of the largest repositories of publicly available biomedical text annotations. In 2018, on average, the repository was accessed by 832 unique users per month.

### 2.6 TextAE: an annotation viewer and editor

TextAE (http://textae.pubannotation.org) is a browser-based visual editor for text annotation. It has following distinguishing features:

1. A visualizer/editor that can be embedded in HTML documents.
2. A REST client capable of obtaining and pushing annotations in a standard RESTful way.
3. A generic tool which is not tightly integrated into a specific implementation of storage.

**Fig. 2.**
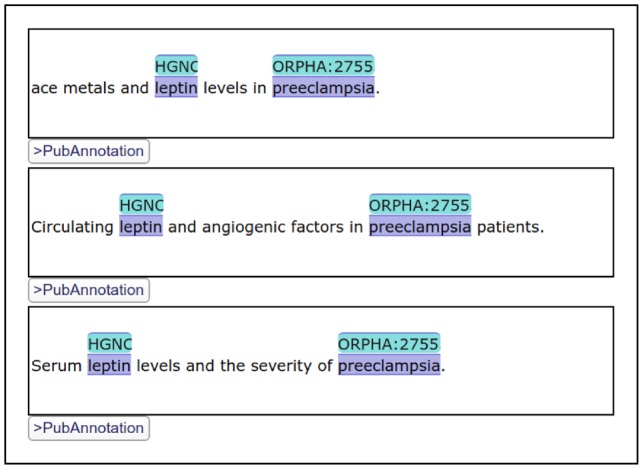
Three TextAE instances which render different annotations: They are parts of the results of the SPARQL search shown in Figure 3. The figure shows the display of identifiers from multiple vocabularies

Among them, the first one is the most important feature, which motivated the development of a new editor while there were already a number of existing web-based annotation editors [e.g. BRAT (Stenetorp *et al.*, 2012), AlvisAE (Papazian *et al.*, 2012)]. By developing a visualizer/editor of text annotation which can be embedded in HTML documents, we intended to improve the reusability of text annotation. Additionally, an instance of TextAE can be embedded in a HTML document as a *div* element. Figure 2 shows three TextAE instances embedded in one document. TextAE works as a REST client to get the annotation from a REST server. So, while annotations are stored in dedicated databases like PubAnnotation, specific portions of them may be freely included in documents in the web. Note that TextAE works not only as a visualizer but also as an editor, so that annotations can be easily edited within the generated visualization. After editing, the edited annotation can be either pushed to a REST server, e.g. PubAnnotation, or downloaded to a local storage. When necessary, authentication is handled by the browser.

**Fig. 3.**
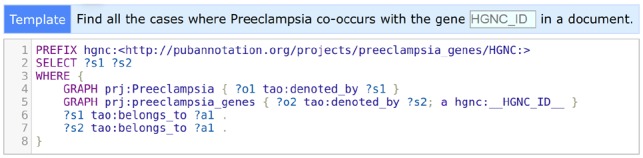
A pre-defined SPARQL template for the *Preeclampsia* project. SPARQL is powerful for searching across datasets and annotation categories, but difficult to learn. PubAnnotation’s support for pre-defined templates allows uses to search for arbitrarily specific entities without learning SPARQL

The SPARQL search interface of PubAnnotation, which was discussed in Section 2.5, uses TextAE to display the results. In case the results of a search are annotations, they are shown in TextAE, so that users can immediately read or sometimes edit them, when necessary. Figure 2 shows a part of the results, 3 out of 410, of the SPARQL query shown in Figure 3.

## 3 Results

The PubAnnotation ecosystem runs as a collection of web services and web clients. Although it has been developed as a versatile platform for text annotation and a repository of shareable resources like annotations and dictionaries, a special focus has been placed on implementing an Agile dictionary-based text annotation platform. Below is a typical scenario of performing dictionary-based text annotation. Note that at each step, the relevant tool, and the section it is described, is written in parenthesis.

1. To prepare a dictionary (PubDictionaries, Section 2.4).
2. To prepare a collection of texts (PubAnnotation, Section 2.5).
3. To annotate the texts using the dictionary (PubAnnotation/PubDictionaries, Section 2.5).
4. To evaluate the results (PubAnnotation/TextAE, Section 2.5).
5. If the evaluation is not satisfactory, improve the dictionary and go back to step 3 (PubDictionaries, Section 2.4).
6. To annotate a large amount of text if necessary using the final dictionary. (PubAnnotation/PubDictionaries, Section 2.5).
7. To search the resulting annotation (PubAnnotation, Section 2.5), or to reuse the annotation (TextAE, Section 2.6).

As discussed in Section 1, all of previous similar efforts have focused on addressing the problem of end-user access to text mining by making it easier for users to interact with the tools. In contrast, the work reported here takes the approach of making it easier for end-users to engage in the annotation process from start to finish, as illustrated the scenario above.

To demonstrate the functionality of the PubAnnotation ecosystem, we present two use cases. Both require dictionary-based annotation as guided by the scenario described above. Note that Steps 3–5 of the workflow may be repeated until the annotation quality is judged satisfactory and that the improvements from this iterative process generate multiple dictionary revisions.

### 3.1 Use Case 1: GlycoEpitope database curation

Glycan analysis has been an important topic of focus within a wide range of life science research and biotechnology. Glycans are carbohydrate sugar chains identified as part of every cell type; they interact with various proteins, viruses, bacteria and antibodies. They are often found on the cell surface, serving as ‘switches’ in toggling various cellular functions (Hakomori, 1984). A large number of polyclonal or monoclonal antibodies have been used to analyze their expression and function. The *GlycoEpitope* database integrates a variety of useful information on carbohydrate antigens and their antibodies (Kawasaki *et al.*, 2006).

To aid in the curation of locational information for glycan epitopes, the PubAnnotation ecosystem was used. First, an annotation dataset, *GlycoBiology-MAT*, was produced in following way:

1. As we were interested in terms for dissection of human organs, the *MAT (Minimal Anatomical Terminology)* ontology was chosen as the most prospective source of relevant vocabulary. On PubDictionaries, a dictionary, *MAT*, was created, into which the labels and synonyms of all the classes from the *MAT* ontology were compiled (*n* = 722).
2. An annotation project, *GlycoBiology-MAT*, was created in PubAnnotation. The PMIDs of all the articles from the journal *GlycoBiology* were collected using PubMed (*n* = 2931). The PMIDs were fed into the project, to populate it with the texts of titles and abstracts of the articles.
3. The texts in the project were annotated using the dictionary *MAT*. Using the initial dictionary, 6635 annotation instances were produced.
4. The annotation result was manually inspected to find false positives (FPs) and false negatives (FNs).
5. The dictionary was revised to remove the FPs and FNs found at Step 4, and the process went back to Step 3 for re-annotation. The loop was continued until the user was satisfied with the annotation. The final dictionary was ended up with 559 entries (The dictionary is available at http://pubdictionaries.org/dictionaries/MAT.), which was then used to produce the final annotation dataset with 5241 annotation instances.

**Fig. 4.**
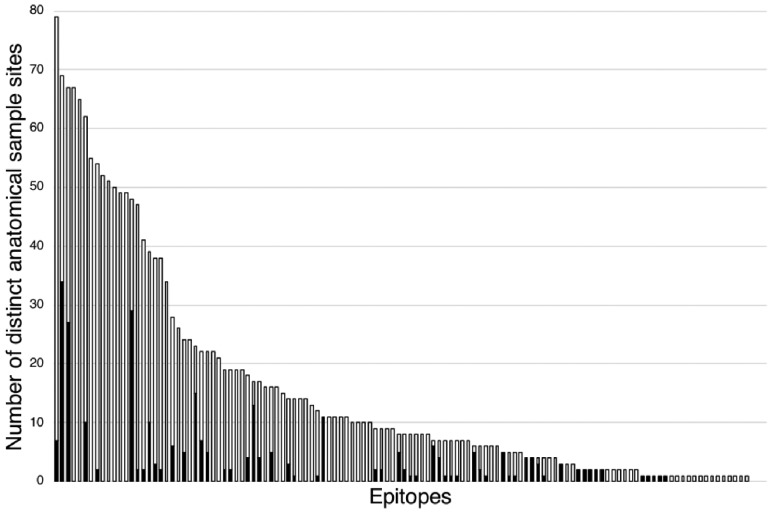
The number of locations per epitope, that are stored in database (indicated by black bars), and that are extracted from literature (white bars). Epitopes are sorted by the number of locations extracted from literature. The difference indicates the potential of further curation through mining the annotation

**Fig. 5.**
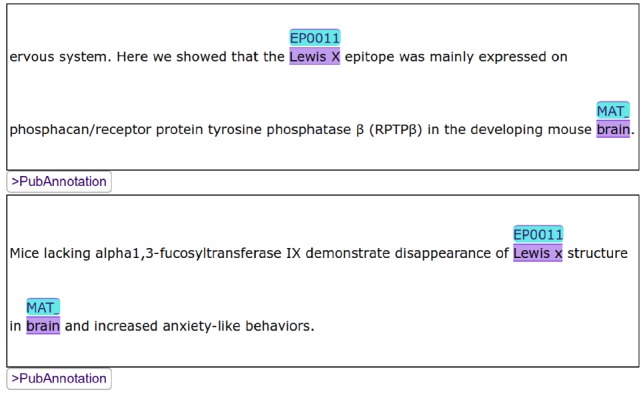
Some indicators of association between Lewis X and brain which are found through the SPARQL search over the annotated data

Another dictionary, *GlycoEpitope*, was created using the entries from the *GlycoEpitope* database. It was then used to produce an annotated dataset, *GlycoBiology-Epitope*. The two datasets were examined using the SPARQL interface of PubAnnotation. In Figure 4, the white bars show the statistics on probable anatomical locations of epitopes indicated by the annotated dataset. The black bars show the statistics on already curated locations of epitopes in the database. The difference indicates the number of candidates for further curation, which are predicted by the annotated dataset. Although the candidates are under reviewing, an initial observation indicates that some candidates are well supported by evidences as exemplified in Figure 5.

### 3.2 Use Case 2: investigating the distribution of literature on Preeclampsia genes

PE is a leading cause of maternal and fetal morbidity and mortality (Backes *et al.*, 2011), with no known cure except delivery of the placenta. Transcriptional profiling of human placenta from pregnancies complicated by PE has been extensively performed as a means to identify differentially expressed genes (DEGs). Unfortunately, the decisions to investigate DEGs experimentally are biased by many factors, which is widely reflected in the literature; the distribution of research on genes is extremely skewed, with the majority of published evidence covering a very small set of genes (Pandey *et al.*, 2014; Riba *et al.*, 2016). To investigate this phenomenon in PE, we annotated publicly available high-throughput transcriptional profiling data from human PE experiments. To accomplish this, we first identified PE-associated DEGs (*n* = 17 322) via meta-analysis of 12 experiments downloaded from the Gene Expression Omnibus. Then, we utilized the PubAnnotation ecosystem to help determine how many of the PE-associated DEGs had literature evidence. First, an annotation dataset, *PE* was produced, in following way:

1. A PubDictionaries dictionary, *PE*, was created, with the three entries, *PE*, *pre-eclamptic toxemia* and *pre-eclamptic toxemia*, which were curated from the *PE* ontology.
2. The PMIDs of all the articles with the MeSH term *PE* were collected using the PubMed interface (*n* = 19 720). The annotation project *PE* was created in PubAnnotation, and it was populated with the texts by feeding the PMIDs to it.
3. The texts in the project were annotated using the initial dictionary *PE*, which produced 51 123 annotation instances.
4. The annotation result was manually inspected. In the initial inspection, lots of FPs were observed, and the following inspection was geared toward finding synonymous terms to *PE*. To find synonyms, the strategy taken was to scan the documents with no annotations, motivated by the assumption that a document with the MeSH term *PE* is likely to contain a synonym of PE if it is not annotated by the term PE itself.
5. The dictionary was revised to incorporate the new synonyms. After the revision, the process went back to Step 3 for re-annotation. After a new term is added, the precision of the term was evaluated in a random sample. The loop was continued until the user was satisfied with the annotation. The final version of the dictionary was ended up with 22 entries, which was then used to produce the final annotation dataset with 68 708 annotation instances.

In a similar way, a second dictionary, *preeclampsia_genes*, was created, using the PE-associated DEGs (names as labels with corresponding HGNC identifiers), which was then used to produce another annotation dataset, *preeclampsia_genes*, in PubAnnotation.

**Fig. 6.**
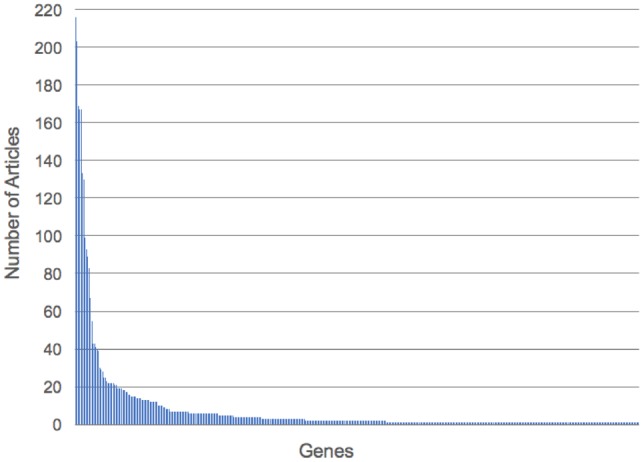
Distribution of articles mentioning PE-associated genes

Of the 17 322 PE-associated DEGs identified from the meta-analysis, 417 had literature evidence in at least 1 article in PubAnnotation. Of these 417 genes, PGF (*n* = 216), TYK2 (*n* = 203), VEGFC (*n* = 169), VEGFA and VEGFB (*n* = 167), INS (*n* = 133) and ENG (*n* = 130) were found to be annotated in the greatest number of articles. In contrast, 45% (*n* = 187) of the PE-associated DEGs were only annotated to a single article. These results are consistent with the literature and suggest a skewed distribution (see Fig. 6) of published literature on PE-associated DEGs.

## 4 Discussion

Bioinformatics occupies an intersectional area that includes problems of ‘pure’ research, software engineering and human–computer interaction. Recent work has demonstrated the potential for tackling some of the core problems of computational biology via the Agile model. Applications have included sequencing (Mishima *et al.*, 2011), metagenomics (Silva *et al.*, 2016) and searching biological networks (Su *et al.*, 2010). As we consider whether Agile methodologies have broader applicability in bioinformatics, text mining offers an especially good test case, because non-deterministic problem spaces are a special challenge for Agile methodologies, and natural language processing (NLP) is a non-deterministic domain par excellence (Chris and Schuetze, 1999). The PubAnnotation ecosystem is an especially revealing test-bed because it poses a known challenge to Agile methodology: distributed development teams. Agile methodologies are that they tend to assume a co-located group of people. But, much of bioinformatics is done by internationally distributed groups (Butte and Chen, 2006), and PubAnnotation in particular is a group effort that crosses continental boundaries (Kim *et al.*, 2015). So, the work discussed here is informative with respect to at least two major challenges for Agile methodologies: non-determinism, and distributed development. What our results suggest about these two issues is that Agile methodologies are a practical approach for bioinformatics.

The limited history of text mining research within an Agile framework is informative with respect to just how large the contributions of this approach could be. There has been just enough prior work on Agile methodologies for text mining to suggest that it is a plausible framework for both research and development. The PubAnnotation ecosystem has extended that research in a number of novel directions. First, the previous work on Agile text mining has taken place in a highly restricted domain: with the exception of prior work on the topic was applied to only a single textual genre, electronic health records (Cormack *et al.*, 2015; Shivade *et al.*, 2014); in contrast, PubAnnotation has been applied to scientific publications as described here, as well as to database contents (Wang *et al.*, 2018). Second, previous work has made a point of separating iterative text mining system development from lexical resource development (Cormack *et al.*, 2015). Although there is certainly an advantage to being able to modularize a system such that functionality is separated from resources, the work reported here maintains that modularity while still facilitating iterative development of lexical resources, entirely separately from—and in addition to—iterative development of text mining functionality. Third, previous work has covered only English data, while PubAnnotation has been applied to other languages [e.g. French (Névéol *et al.*, 2014, Japanese, Chinese and Korean, as well, in order to test generalizability]. Fourth and perhaps most importantly for the broader scientific community, the previous work on Agile text mining of which we are aware has focused on a proprietary software package (Cormack *et al.*, 2015; Shivade *et al.*, 2014; Sukkarieh and Kamal, 2009). In contrast, PubAnnotation is completely open-source, with all of the benefits for reproducibility, replicability and transparency that it brings.

As discussed in Section 1, the PubAnnotation ecosystem with a focus on dictionary-based annotation and its two use cases were presented to demonstrate the importance of ‘customizability’ as a feature of text annotation system, and to promote the concept of ‘Agile’ text mining. For every step in the typical scenario of dictionary-based annotation, which is shown in the Section 3, the PubAnnotation ecosystem provides easy-to-use interface, in both web UIs and programable RESTful APIs, making operation of tools for each step a trivial task.

For both use cases, customization was made to the dictionaries. In each use case, the user began with a dictionary of reasonable choice at the time: the most prospective existing resource was chosen to initialize the dictionaries. In both cases, however, the annotation results using the initial dictionaries were not satisfactory, and the users needed to customize the dictionaries. For each use case, the customization process (which corresponds the Steps 3–5) was the prevailing steps of the entire process, and it was a half-day effort in a hackathon environment (http://2017.biohackathon.org). For the use Case 1, the customization loop was conducted toward reducing noisy annotation. As the result, the noisy annotation was reduced by 21% (from 6635 to 5241). For use Case 2, the customization loop was conducted toward improving the coverage of annotation. As the result, the coverage was improved by 34% (from 51 123 to 68 708). For both use cases, evaluation of the quality of annotation was determined subjectively by the users, which reflects the goal of the work: to enable users to get annotation for their own purpose as quickly as possible. PubAnnotation also provides a powerful search interface to examine the annotation, even across multiple datasets. For reproducibility, the ecosystem also supports sharing all the dictionaries and annotations. Curators of the GlycoEpitope database evaluated the annotation, which was produced in half a day, potentially to be useful for substantially reducing the curation process, which takes 3–5 h on average to curate one antibody.

Comparing PubAnnotation to other user-facing annotation systems is instructive for considering both the strengths and the weaknesses of the platform. One such system is Argo. A strength of Argo is that it allows the integration of manual annotation into otherwise automatic text processing pipelines (Batista-Navarro *et al.*, 2016; Rak *et al.*, 2012). This is important because manual annotation is a classic example of a task that is suitable for domain experts, as opposed to language processing people (Stubbs, 2013).So, from an Agile text mining perspective, manual annotation can be thought of as the textual equivalent of a user story. This has strengths for the development of corpora.

Another user-facing system is Textpresso. Where Argo’s strengths lie mainly in support for the creation of corpora, the Textpresso system (Müller *et al.*, 2004, 2018) is a highly adaptable platform for the curation of model organism databases. This is important because model organism databases have been major enablers of progress in genomics research, the scientific literature is the major source for the curation of those databases, and use of text mining in that curation work is not practical without user-driven development of interfaces (Wang *et al.*, 2016)—a classic application of Agile methodology. Its strength in this area is illustrated by the many organism-centric systems that have been based on it (Van Auken *et al.*, 2012). Textpresso is highly customizable at the organizational level, allowing for the incorporation of multiple biomedical ontologies. From an Agile perspective, the ability to easily incorporate new knowledge sources can be seen as exemplary of the capacity to be flexible and responsive to customer-requested changes.

With that context established, the two use cases described above can be better understood in terms of their relevance to illustrating how the PubAnnotation platform allows for implementation of Agile processes in text mining efforts: it combines the user story support of Argo with the flexible responsiveness to customer needs of the Textpresso family of curation tools. Additionally, its use of Semantic Web technology allows it to support sharing and collaboration, which are difficult for Agile methodologies in general. Furthermore, its large storage capacity allows for the storage of multiple versions of the data that is output at intermediate steps of a NLP pipeline, which facilitates the reproducibility and replicability of the research that it supports.

## 5 Conclusion

The staggering volume of scientific literature produced each year provides an unprecedented opportunity for scientific discovery. But, for most researchers, this amount of information is simply unmanageable. Text mining shows promise for providing researchers with a wide variety of tools, applications, and annotated corpora. However, fundamental questions remain regarding the utility of existing tools and resources for addressing the specific needs of biologists and or bioinformaticians.

Here, we present the PubAnnotation ecosystem, an open, Agile text mining framework purposefully designed to engage users throughout the entire annotation process. It both reformulates the use of text mining by end-users from a task of building text mining systems to a task of retrieving annotations that have already been done, and makes it easier for non-specialists to adapt a text mining system to their own needs by using the pre-existing PubAnnotation ecosystem. Although this article focuses on discussing dictionary-based approaches to do that adaptation, the PubAnnotation ecosystem also includes machine learning-based annotators, opening the opportunity to use them in combination with the dictionary-based approaches. Also, many machine learning-produced annotations are already included in the repository.

Existing mainstream biomedical text mining approaches emphasize performance optimization on large volumes of documents, utilize benchmark annotations, and apply proxy figures of merit (e.g. the F-measure). Although it is clear that such approaches have contributed to the advancement of text mining technology from a technical perspective, it also seems clear from work like that of (Alex *et al.*, 2008; Wang *et al.*, 2016) that these approaches fail to satisfy scientists who want understand the underlying processes that they perform. We hope that this work will help to solve these issues, while providing biologists and bioinformaticians with a means to accomplish their text mining goals.

Although the two uses cases presented in the Results section demonstrate how easily text annotation data can be obtained and customized, much room for improvement remains, particularly with the loop for manual inspection of annotation. It is now largely left to the empirical decisions of the users who develop the dictionaries. The future looks bright for text mining in the hands of biomedical researchers.
